# A population-based study of familial coaggregation and shared genetic etiology of psychiatric and gastrointestinal disorders

**DOI:** 10.1038/s43856-024-00607-7

**Published:** 2024-09-19

**Authors:** Yi-Jiun Pan, Mei-Chen Lin, Jyh-Ming Liou, Chun-Chieh Fan, Mei-Hsin Su, Cheng-Yun Chen, Chi-Shin Wu, Pei-Chun Chen, Yen-Tsung Huang, Shi-Heng Wang

**Affiliations:** 1 Department of Microbiology and Immunology, School of Medicine, College of Medicine, China Medical University, Taichung, Taiwan; 2 National Center for Geriatrics and Welfare Research, National Health Research Institutes, Zhunan, Taiwan; 3 Division of Gastroenterology and Hepatology, Department of Internal Medicine, National Taiwan University Hospital, Taipei, Taiwan; 4 Department of Internal Medicine, National Taiwan University College of Medicine, Taipei, Taiwan; 5 Department of Medicine, National Taiwan University Cancer Center, Taipei, Taiwan; 6 Center for Population Neuroscience and Genetics, Laureate Institute for Brain Research, Tulsa, OK USA; 7 Department of Radiology, School of Medicine, University of California San Diego, La Jolla, CA USA; 8 Department of Public Health, College of Public Health, China Medical University, Taichung, Taiwan; 9 Department of Psychiatry, Virginia Institute for Psychiatric Behavioral Genetics, Virginia Commonwealth University, Richmond, VA USA; 10 Department of Psychiatry, National Taiwan University Hospital, Yunlin branch, Douliu, Taiwan; 11 Institute of Statistical Science, Academia Sinica, Taipei, Taiwan; 12 Department of Medical Research, China Medical University Hospital, China Medical University, Taichung, Taiwan

**Keywords:** Gastroenterology, Genetic association study

## Abstract

**Background:**

It has been proposed that having a psychiatric disorder could increase the risk of developing a gastrointestinal disorder, and vice versa. The role of familial coaggregation and shared genetic loading between psychiatric and gastrointestinal disorders remains unclear.

**Methods:**

This study used the Taiwan National Health Insurance Research Database; 4,504,612 individuals born 1970–1999 with parental information, 51,664 same-sex twins, and 3,322,959 persons with full-sibling(s) were enrolled. Genotyping was available for 106,796 unrelated participants from the Taiwan Biobank. A logistic regression model was used to examine the associations of individual history, affected relatives, and polygenic risk scores (PRS) for schizophrenia (SCZ), bipolar disorder (BPD), major depressive disorder (MDD), and obsessive-compulsive disorder (OCD), with the risk of peptic ulcer disease (PUD), gastroesophageal reflux disease (GERD), irritable bowel syndrome (IBS), and inflammatory bowel disease (IBD), and vice versa.

**Results:**

Here we show that parental psychiatric disorders are associated with gastrointestinal disorders. Full-siblings of psychiatric cases have an increased risk of gastrointestinal disorders except for SCZ/BPD and IBD; the magnitude of coaggregation is higher in same-sex twins than in full-siblings. The results of bidirectional analyses mostly remain unchanged. PRS for SCZ, MDD, and OCD are associated with IBS, PUD/GERD/IBS/IBD, and PUD/GERD/IBS, respectively. PRS for PUD, GERD, IBS, and IBD are associated with MDD, BPD/MDD, SCZ/BPD/MDD, and BPD, respectively.

**Conclusions:**

There is familial coaggregation and shared genetic etiology between psychiatric and gastrointestinal comorbidity. Individuals with psychiatric disorder-affected relatives or with higher genetic risk for psychiatric disorders should be monitored for gastrointestinal disorders, and vice versa.

## Plain language summary

It has been proposed that people with psychiatric disorders such as depression could have an increased chance of developing gastrointestinal disorders such as irritable bowel syndrome. We looked at whether this was the case in a large number of people from Taiwan. We found that people with a psychiatric disorder, or with relatives having a psychiatric disorder, were more likely to have gastrointestinal disorders, and vice versa. These findings suggest that people who have psychiatric disorders or have psychiatric disorder-affected relatives should be monitored for gastrointestinal disorders, and vice versa, to enable them to benefit from all the treatments they might need to improve their health.

## Abstract (web summary)

Pan et al. examine whether there is a shared pathophysiological mechanism underlying brain-gut comorbidity. This population-based cohort and biobank study demonstrates that there is familial coaggregation and shared genetic etiology between psychiatric and gastrointestinal comorbidity.

## Introduction

Accumulating evidence from observational studies suggests that psychiatric disorders could increase the risk of gastrointestinal disorders, and vice versa^1–25^. However, existing epidemiological literature has focused on few pairs of psychiatric and gastrointestinal disorders; a comprehensive assessment of the brain-gut comorbidity is warranted. In addition to bidirectional causality behind the brain-gut comorbidity evidenced from Mendelian randomization analyses^26,27^, the comorbidity may be due to a shared genetic etiology.

Exploring the extent of familial coaggregation between the two disorders may provide insights into the underlying etiology of their comorbidities that could imply shared genetic loadings or environmental factors. A family history of psychiatric disorders was associated with irritable bowel syndrome (IBS)^28^; individuals with parental IBS and inflammatory bowel disease (IBD) have a higher risk of major depressive disorder (MDD)^29^, and autism, respectively^30^. Examining the extent of familial coaggregation in relatives with varying degrees of genetic similarity and comparing the strength of recurrence association could provide further insights^23,31^. We expect that the association would be greater in closer relatives and the risk would be higher in individuals with more affected relatives, which would support the existence of shared genetic background for comorbidity.

Genome-wide association studies (GWAS) have identified the susceptibility loci for psychiatric disorders^32–37^ and gastrointestinal disorders, and these large-scale GWASs have been performed in populations of European majority^26,38–45^. Based on GWAS summary, a significant positive genetic correlation exists between psychiatric and gastrointestinal disorders^26,27,43,45,46^ and several shared susceptibility loci have been identified with pleiotropic effects^26,40,45,47,48^ in European populations. Although the efforts to understand the genetic mechanism behind the brain-gut comorbidity are promising, these results based on predominantly molecular genetic studies may be affected by extreme sampling of cases and thus require more replication. However, to our knowledge, no population-based familial study comparing varying degrees of genetic similarity with sufficient statistical power has evaluated the issue. In addition, culture behaviors could vary across populations, and the genetic correlations between human complex traits may vary across populations and cultures^49^. Although the genetic correlation behind the comorbid relationship between psychiatric and gastrointestinal diseases has been proposed in European populations using summary statistics, whether it could be replicated in different cultured populations remained unclear.

This study aimed to investigate comorbidity, familial coaggregation and shared genetic loading between four common psychiatric disorders in adults with publicly available GWAS summary and good validity in health insurance databases, including schizophrenia (SCZ), bipolar disorder (BPD), MDD, and obsessive-compulsive disorder (OCD), and four common gastrointestinal disorders with publicly available GWAS summary, including peptic ulcer disease (PUD), gastroesophageal reflux disease (GERD), IBS, and IBD, by performing a population-based cohort study and biobank study. First, we explored within-individual comorbidity and within-family coaggregation, in which relatives with varying degrees of genetic similarity were considered, using data from Taiwan’s single-payer compulsory insurance program. Second, we explored the association of polygenic risk score (PRS)^50^ for psychiatric disorders with gastrointestinal disorders, and vice versa, among a large collection of genome-wide genotyping data of community participants from the Taiwan Biobank. Third, we explored the genetic correlation between psychiatric and gastrointestinal disorders using GWAS summary statistics. We also explored bidirectional causal associations (vertical pleiotropy) between psychiatric and gastrointestinal disorders by Mendelian randomization analyses.

Here we show that there is familial coaggregation and shared genetic etiology between psychiatric and gastrointestinal comorbidity. Individuals with psychiatric disorder-affected relatives or with higher genetic risk for psychiatric disorders should be monitored for gastrointestinal disorders, and vice versa.

## Methods

We conducted a nationwide cohort study using the National Health Insurance Research Database (NHIRD) and a molecular genetic study using the Taiwan Biobank, linking the genotyping information to NHIRD. The study described in this paper was approved by the Institutional Review Board (IRB) of the Central Regional Research Ethics Committee of the China Medical University, Taichung, Taiwan (CRREC-108-30); the requirement for informed consent to have been obtained from all participants of the NHIRD whose data was included was waived because the NHIRD contains de-identified data.

The National Health Insurance (NHI) of Taiwan, a single-payer health insurance system, was initiated in 1995 and covers more than 99% of the Taiwanese population. The NHI’s claims data are released as the NHIRD, held by the Ministry of Health and Welfare, Taiwan. Informed consent was not obtained from people whose data is included in the NHIRD because the data is de-identified. Before requesting access to the NHIRD for research purposes, researchers must receive an approval from the IRB. The IRB and the Ministry of Health and Welfare, Taiwan approved our application to access the NHIRD.

The data collection of Taiwan Biobank was approved by the Ethics and Governance Council of Taiwan Biobank and the Ministry of Health and Welfare, Taiwan. Taiwan Biobank obtained informed consent from all participants for research use of the collected data and samples and the linkage with the NHIRD. The IRB, the Ministry of Health and Welfare, Taiwan, and the Taiwan Biobank approved our application to access the cross-linkage data of Taiwan Biobank and NHIRD.

### Cohort study subjects and measurements

We integrated several databases from the NHIRD. Registry for beneficiaries contains information on the identifiers of the relationships between insured person and their dependents. Only spouses and blood relatives are eligible as dependents of an insured person. We used the covered-insurance relationship to infer pedigree relationships, and the accuracy of the inferred pedigree is excellent^51^. Using the Registry for Beneficiaries, as described by Wang et al.^51^, family information was ascertained based on the information of the identifiers and unique personal identifier numbers of the parent, child, grandparent, grandchild, and spouse. Indirect identification of the pedigree was further performed based on the above-mentioned direct identification. We selected families wherein the parent and child had an age difference of at least 12 years to maximize the probability to obtain correct familial relatedness. Siblings were defined as two individuals having the same parents. Twins were defined as two individuals being born on the same day and from the same parents; however, monozygotic and dizygotic twins could not be distinguished. Triplets were not included in this study. Death certifications were used to follow up the vital status of the study population. Inpatient and outpatient claims data were used to identify disease diagnoses.

We identified psychiatric and gastrointestinal disorders using inpatient and outpatient claims data from 1998 to the date of death or until the end of 2020. Detailed information for identifying psychiatric and gastrointestinal disorders on the International Classification of Diseases codes is presented in Supplementary Table 1. At least two outpatient or one inpatient admission for a disease were considered, to increase diagnostic precision. Comorbidity was defined if having more than one disease during the follow-up duration.

### Biobank study subjects and measurements

The Taiwan Biobank^52,53^, the largest government-supported biobank in Taiwan since 2012, recruits community-based participants aged 30–70 years with no history of cancer. Each participant provided blood samples and attended face-to-face interviews.

This study comprised 131,048 individuals for whom genome-wide genotyping was carried out using the custom Taiwan Biobank chips ran on the Axiom Genome-Wide Array Plate System (Affymetrix, Santa Clara, CA, USA); 27,716 participants were genotyped on theTWBv1 chip and 103,332 participants genotyped on theTWBv2 chip. Quality control was performed for the two batches separately before imputation, including the exclusion of variants with a call rate <5%, minor allele frequency <0.001, and deviation from Hardy-Weinberg equilibrium with *p* < 1E-06. As described in our prior paper^54^, we used the 504 EAS panel from 1000 Genomes Project^55^ and the 973 TWB panel from whole-genome sequencing in TWB participants as the reference panel to impute the genotypes with IMPUTE2 for the two batches separately (16,537,409 variants for TWBv1 batch and 16,211,759 variants for TWBv2 batch) and then retained variants with imputation info > 0.7 (13,803,412 variants for TWBv1 batch and 13,572,189 variants for TWBv2 batch). A total of 12,601,684 variants were available in both batches and kept for subsequent PRS calculation.

As described in our prior paper^54^, duplicated samples, non-EAS samples, as well as samples with a missing rate of more than 2%, or heterozygosity outliers (exceeding 5 standard deviations) were excluded, and 128,775 samples remained. Principal components analysis was performed to account for population stratification, and top 20 principal components (PCs) were included in the model for adjustment. To remove cryptic relatedness, we estimated the identity by descent (IBD) sharing coefficients (PI-HAT = probability (IBD = 2) + 0.5 × Probability (IBD = 1)) between any two participants. For pair-wise participants with PI-HAT > 0.1875, one of the study participants was removed; 106,806 unrelated participants were kept. Finally, 106,796 unrelated participants were successfully linked to NHIRD to retrieve their disease diagnoses and included in the subsequent PRS analysis.

We derived PRS for four psychiatric and four gastrointestinal disorders using PRS-CSx (if large-scale GWAS summary was available in both European and East Asian populations)^56^, or PRS-CS^57^. The PRS was normalized to a Z-score and categorized into deciles for ease of interpretation.

Data from the Psychiatric Genomics Consortium (PGC) meta-analysis were used as a discovery sample to identify the risk variants for SCZ, BPD, MDD, and OCD, and the PRS for each corresponding psychiatric disorder was calculated in the Taiwan Biobank. To optimize the PRS prediction, we derived PRS for SCZ using PRS-CSx^56^, a state-of-the-art Bayesian polygenic modeling method jointly modeling GWAS summary statistics of different discovery samples, to integrate GWAS in 22778 cases and 35362 controls of East Asian descent^35^ and GWAS in 53,386 cases and 77,258 controls of European descent^37^, which lead to a better prediction than the PRS derived from GWAS of 67,390 cases and 94,015 controls of European descent majority^37^ in our target samples. We derived PRS for MDD using PRS-CSx to integrate GWAS in 13,042 cases and 88,467 controls of East Asian descent^33^ and GWAS in 246363 cases and 561190 controls of European descent^34^, which led to a better prediction than the PRS derived from the European GWAS only. The PRS for BPD was derived from a discovery sample of 41,917 cases and 371,549 controls of European descent^36^ using PRS-CS^57^, a polygenic prediction method inferring posterior effect sizes of susceptibility variants by utilizing a high-dimensional Bayesian regression framework and continuous shrinkage priors on susceptibility variant effect sizes. The PRS for OCD was derived from a discovery sample of 2688 cases and 7037 controls of European descent^32^ using PRS-CS.

The PRS for PUD was derived from a discovery sample of 16,666 cases and 439,661 controls of European descent^26^ using PRS-CS. The PRS for GERD was derived from a discovery sample of 78,707 cases and 288,734 controls of European descent^43^ using PRS-CS. The PRS for IBS was derived from a discovery sample of 53,400 cases and 433,201 controls of European descent^45^ using PRS-CS. The PRS for IBD was derived from a discovery sample of 25,042 cases and 34,915 controls of European descent^44^ using PRS-CS.

Covariates considered in the biobank study included BMI, education attainment, lifestyles, and diet habits. Educational attainment was classified as elementary school and below, junior high school, senior high/vocational school, university/college, and master and above. Lifestyles included lifetime regular smoking (persisted > 6 months), lifetime regular drinking (>150 cc weekly, persisting for >6 months), and exercise habits. Diet habits included tea and coffee consumption, vegetarian, and late-night supper.

### Statistics and Reproducibility

The distribution of demographic factors, psychiatric disorders, and gastrointestinal disorders was described by numbers and percentages. Generalized estimating equations with an exchangeable working correlation structure were used to consider the non-independence of data within the family cluster in all analyses. A generalized linear model with binomial distribution and logistic link function was used to estimate the adjusted ORs and 95% CIs.

To evaluate the within-individual association, in which individuals with a psychiatric disorder were more likely to have comorbid gastrointestinal disorders compared with population controls, and vice versa, sex, birth cohort, age (length of follow-up), income level, and urbanization level were adjusted.

To investigate familial coaggregation between psychiatric and gastrointestinal disorders, we examined whether individuals with affected relatives, including parents, same-sex twins, and full siblings, with a psychiatric disorder are more likely to have gastrointestinal disorders than individuals with unaffected relatives, and vice versa.

Parental history of psychiatric/gastrointestinal disorder was categorized as both parents without disease history (P−M−, reference group), maternal history only (P−M+), paternal history only (P+M−), and both parents with disease history (P+M+). To evaluate the strength of the association between parental psychiatric/gastrointestinal disorders and those of the children, the first adjustment model included sex, birth cohort, age, income level, urbanization level, father’s age, and mother’s age. The second adjustment model further included the outcome individual’s self-corresponding psychiatric/gastrointestinal diagnosis.

Regarding the history of psychiatric/gastrointestinal disorder in full siblings, two categorized methods were applied; first, any sibling with the disease vs. no disease (reference group); second, no disease (reference group), 1, and ≥ 2 siblings with the disease to explore the dose-response relationship. To evaluate the strength of sibling coaggregation, the first adjustment model included sex, birth cohort, age, income level, urbanization level, sibling’s age, and sibling size, while the second adjustment model further included the outcome individual’s self-corresponding psychiatric/gastrointestinal diagnosis. To evaluate the strength of the coaggregation within same-sex twins, the adjustment was similar, except for sibling age and size. A higher OR in same-sex twins than in full siblings provides evidence for shared genetic influences on the coaggregation of psychiatric and gastrointestinal disorders.

Sex-stratified analyses for within-individual association and within-family associations were estimated to explore sex differences.

To study shared polygenetic liability, we tested the association of PRS for psychiatric disorders with gastrointestinal disorders, and vice versa, among 106,796 unrelated individuals. The significance of each PRS was evaluated using logistic regression models adjusted for sex, age, batch version, and the first 20 principal components^58,59^. To evaluate the robust of the PRS association, several potential confounders were further adjusted; first, BMI, education attainment, and lifestyles were additionally adjusted, and second, diet habit was further adjusted. Dummy variable adjustment was used for handling missing data. To further explore the pure effect of PRS, individuals with a corresponding psychiatric/gastrointestinal disorder were excluded from PRS testing. A total of 106,127; 105,071; 92,828; and 106,276 eligible individuals were tested for PRS for SCZ; BPD; MDD; and OCD, respectively, while 61,571; 74,104; 91,739; and 104,151 eligible individuals were tested for PRS for PUD; GERD; IBS; and IBD, respectively. Sex differences in the PRS association were tested.

Linkage disequilibrium (LD) score regression^60,61^ using European reference panel was used to estimate the genetic correlation between psychiatric and gastrointestinal disorders.

Bidirectional Mendelian randomization analyses were performed to examine the causality between psychiatric and gastrointestinal disorders. To extract independent variants, LD clumping was conducted based on *r*^2^ > 0.0001 within a 1000 kb window. Variants with genome-wide significance (*p* < 5 × 10^−8^) for exposure were selected as genetic instruments. The fixed-effect inverse variance weighted method^62^ was considered as main analysis. Several Mendelian randomization methods were used for the sensitivity analysis, including MR-Egger^63^, weighted median^64^, weighted mode^65^, and MR PRESSO^66^.

The significance level was set at 0.05. Considering 16 testing scenarios (four psychiatric disorders times four gastrointestinal disorders), the significance level was set at a Bonferroni-corrected 0.003125. All statistical analyses were performed using SAS statistical package (version 9.4, SAS Institute Inc., Cary, NC, USA).

### Reporting summary

Further information on research design is available in the Nature Portfolio Reporting Summary linked to this article.

## Results

### Cohort description

Altogether 4,504,612 eligible individuals from 2,536,631 families born between January 1, 1970, and December 31, 1999, with information regarding both parents, were included in this study, and they were followed up from 1998 to 2020 to examine individual associations and parental associations. After excluding 10,325 triplets, 13,296 opposite-sex twins, and 1,106,368 single children, 51,664 same-sex twins (monozygotic and dizygotic twins could not be distinguished) and 3,322,959 full sibling(s) from 1,409,874 families were enrolled.

The distribution of demographic characteristics, psychiatric disorders, and gastrointestinal disorders is presented in Supplementary Table 2. The proportion of men in the total cohort was 52.9%. The prevalence of PUD, GERD, IBS, and IBD were 12.6%, 13.1%, 5.8%, and 3.1%, respectively. The prevalence of SCZ, BPD, MDD, and OCD were 0.9%, 1.1%, 6.1%, and 0.5%, respectively. Sex-stratified prevalence is shown in Supplementary Table 3.

### Within-individual and within-family associations

**Fig. 1:**
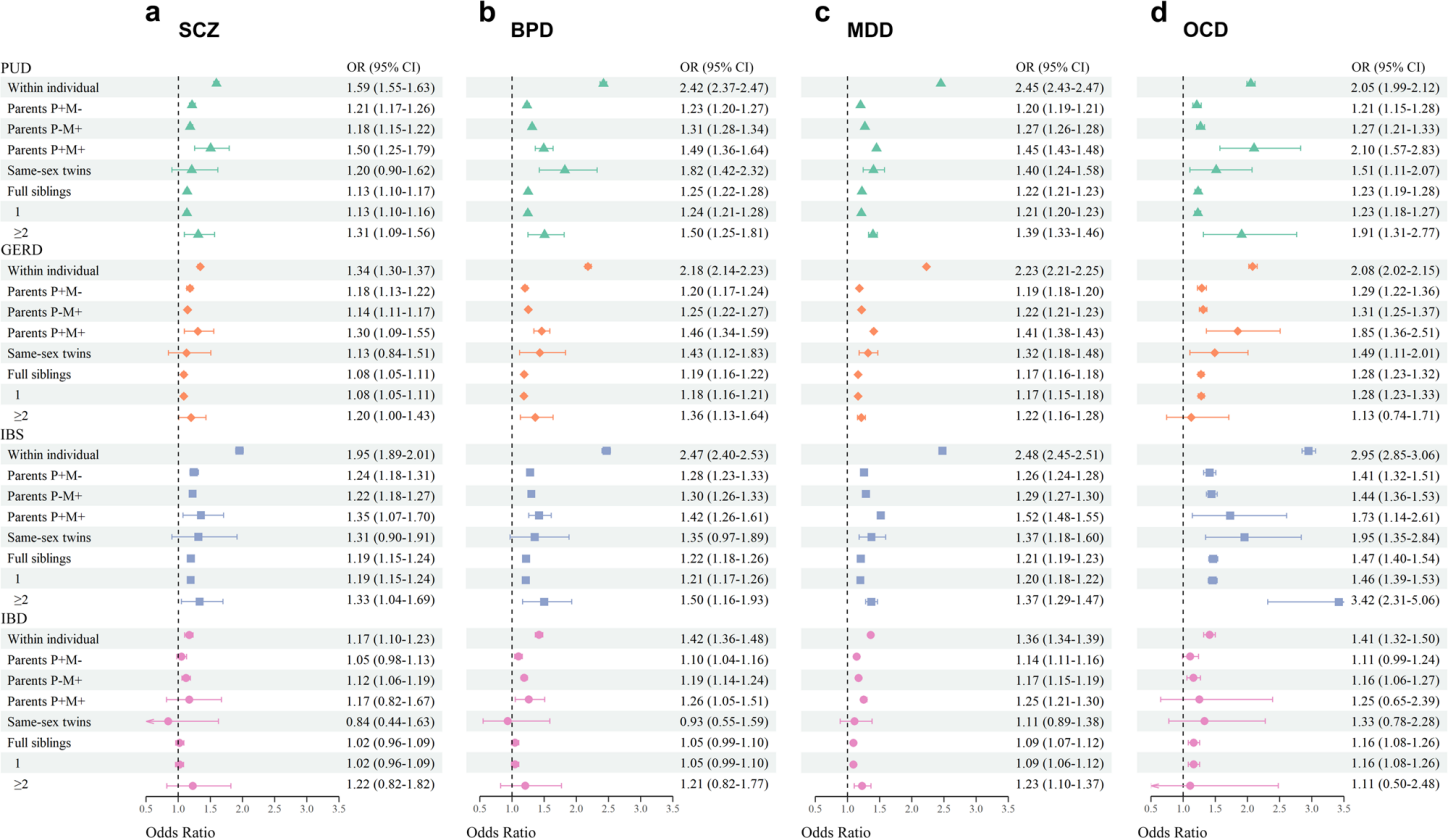
Individual history and family history, including parents, same-sex twins, and full siblings, of psychiatric disorders and the risk of gastrointestinal disorders. **a** schizophrenia (SCZ). **b** Bipolar disorder (BPD). **c** Major depressive disorder (MDD). **d** Obsessive-compulsive disorder (OCD). Odds ratio and 95% confidence interval represents the association estimated in the within-individual analyses and in within-family analysis of different types of relatives. Green triangle indicates peptic ulcer disease (PUD), orange diamond indicates gastroesophageal reflux disease (GERD), blue square indicates irritable bowel syndrome (IBS), and pink circle indicates inflammatory bowel disease (IBD). The sample size for analyses of individual history and parental history = 4,504,612, same-sex twins = 51,664, and full siblings = 3,322,959.

The distribution of comorbidities of psychiatric and gastrointestinal disorders in the total cohort is presented in Supplementary Table 4. Individuals with a psychiatric disorder had increased comorbidity with gastrointestinal disorders, and the odds ratio (OR) ranged from 1.2 to 2.9 (Fig. 1, detailed number in Supplementary Table 4).

The distribution and associations of the family history of psychiatric disorders and risks of gastrointestinal disorders for parents/same-sex twins/full siblings are detailed in Supplementary Data 1–4. After further adjusting for the outcome individual’s self-psychiatric diagnosis, the OR attenuated slightly and remained similar, implying that within-family association was not predominantly attributable to the within-individual association. The ORs for the outcome person’s gastrointestinal disorders across the family history of psychiatric disorders in different relatives are plotted in Fig. 1.

Generally, parental history of psychiatric disorders was associated with a higher risk for gastrointestinal disorders, and the magnitude of the association for P+M+ (OR, 1.2–2.1) was higher than that for P+M−/P−M+ (OR, 1.1–1.5).

Full sibling of SCZ patients had an increased risk of PUD, GERD, and IBS (OR = 1.13, 1.08, and 1.19, respectively). The magnitude of association for those with ≥ 2 SCZ-affected siblings was higher than those with one SCZ-affected sibling.

Full siblings of BPD patients had an increased risk of PUD, GERD, and IBS (OR = 1.25, 1.19, and 1.22, respectively) in a dose-response manner, while same-sex twins (OR = 1.82, 1.43, and 1.35, respectively) had a greater association than full siblings.

Full siblings of patients with MDD had an increased risk of PUD, GERD, IBS, and IBD (OR = 1.22, 1.17, 1.21, and 1.09, respectively), with a dose-response relationship between the number of MDD-affected siblings and the risk of four gastrointestinal disorders. Same-sex twins (OR = 1.40, 1.32, and 1.37 for PUD, GERD, and IBS, respectively) had a greater association than full siblings.

Same-sex twins of patients with OCD had an increased risk of PUD, GERD, and IBS (OR = 1.51, 1.29, and 1.95, respectively); full siblings of patients with OCD had an increased risk of PUD, GERD, IBS, and IBD (OR = 1.23, 1.28, 1.47, and 1.16, respectively), and in a dose-response manner for PUD and IBS.

### Bi-directional analyses

**Fig. 2:**
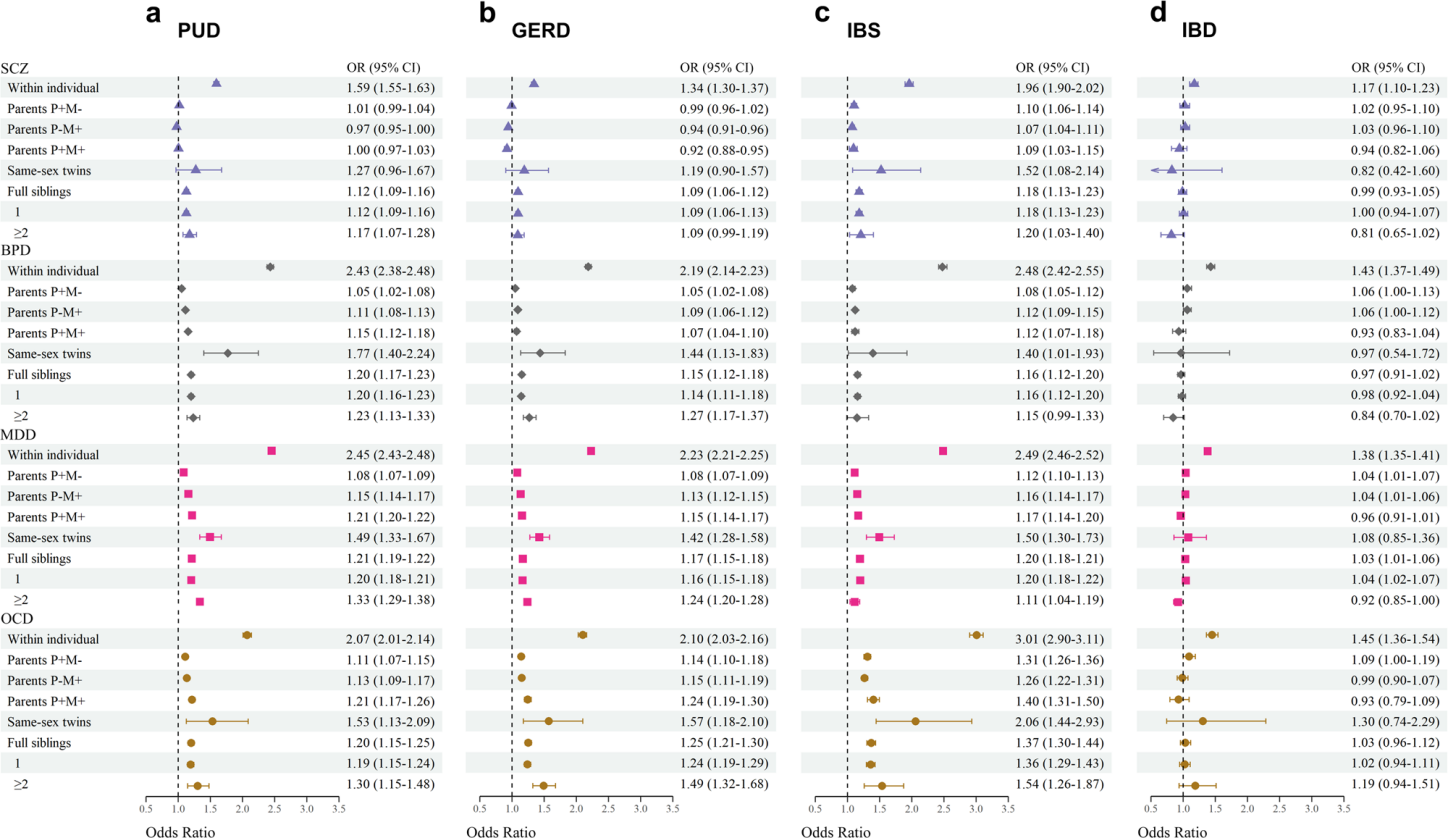
Individual history and family history, including parents, same-sex twins, and full siblings, of gastrointestinal disorders and the risk of psychiatric disorders. **a** Peptic ulcer disease (PUD). **b** Gastroesophageal reflux disease (GERD). **c** Irritable bowel syndrome (IBS). **d** Inflammatory bowel disease (IBD). Odds ratio and 95% confidence interval represent the association estimated in the within-individual analyses and in within-family analysis of different types of relatives. Purple triangle indicates schizophrenia (SCZ), gray diamond indicates bipolar disorder (BPD), pink square indicates major depressive disorder (MDD), and brown circle indicates obsessive-compulsive disorder (OCD). The sample size for analyses of individual history and parental history = 4,504,612, same-sex twins = 51,664, and full siblings = 3,322,959.

Conversely, the associations between the history of gastrointestinal disorders and risks of psychiatric disorders for self/parental/same-sex twins/full siblings are presented in Fig. 2 (detailed in Supplementary Table 5 and Supplementary Data 5–8). Parental PUD and GERD were associated with BPD/MDD/OCD (OR, 1.1–1.2) but not with SCZ. Parental IBS was associated with SCZ/BPD/MDD (OR, 1.1–1.2) and OCD (OR, 1.3–1.4). Parental IBD was not associated with any psychiatric disorder. The bidirectional results for same-sex twins and full siblings were similar (Figs. 1, 2).

### Sex-stratified analyses

Sex-stratified within-individual and within-family associations of psychiatric disorders with the risk of gastrointestinal disorders are presented in Supplementary Fig 1 (detailed numbers in Supplementary Data 9). Generally, the sex-specific estimates for within-family associations were similar. The sex-stratified association of gastrointestinal disorders with the risk of psychiatric disorders is presented in Supplementary Fig. 2 (detailed numbers in Supplementary Data 10).

### Individual-level PRS association in biobank study

The distribution of demographic characteristics and psychiatric and gastrointestinal disorders among the 106,796 biobank participants is shown in Supplementary Table 6. The PRS for psychiatric disorders (2.05%, 0.31%, 0.55%, and 0.04% for SCZ, BPD, MDD, and OCD, respectively) explained a higher variance in the corresponding disease status compared to the PRS for gastrointestinal disorders (0.04%, 0.23%, 0.08%, and 0.01% for PUD, GERD, IBS, and IBD, respectively) (Supplementary Table 7).

**Fig. 3:**
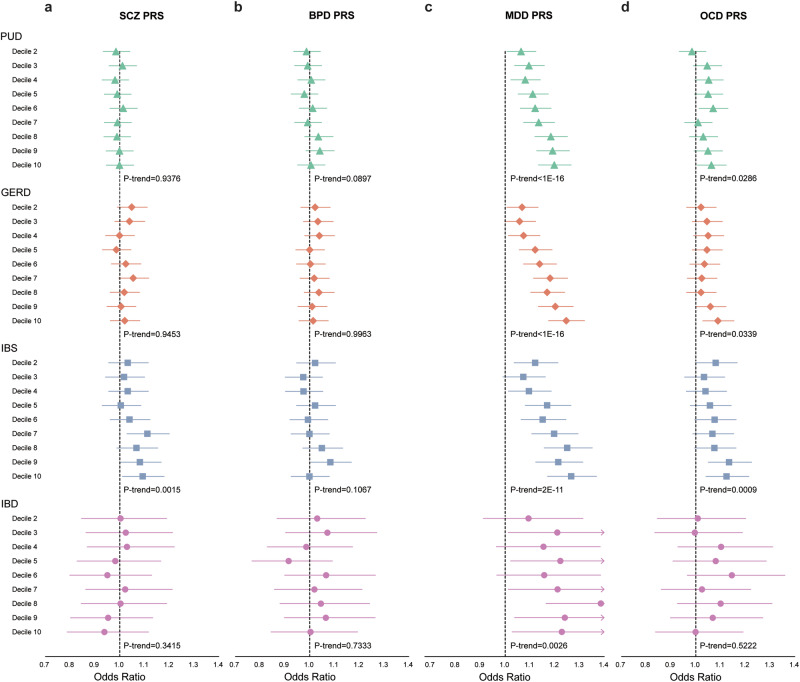
Association of decile of polygenic risk score (PRS) for psychiatric disorders with gastrointestinal disorders. **a** Schizophrenia (SCZ). **b** Bipolar disorder (BPD). **c** Major depressive disorder (MDD). **d** Obsessive-compulsive disorder (OCD). Odds ratio and 95% confidence interval for the highest nine deciles, respectively, compared to the lowest decile. Green triangle indicates peptic ulcer disease (PUD), orange diamond indicates gastroesophageal reflux disease (GERD), blue square indicates irritable bowel syndrome (IBS), and pink circle indicates inflammatory bowel disease (IBD). Sample size = 106,796.

The results of the association of PRS for psychiatric and gastrointestinal disorders are shown in Supplementary Table 8. SCZ PRS was positively associated with IBS (OR per standard deviation (SD) increase in PRS: 1.03, 95% confidence interval (CI): 1.01–1.05, *p* = 0.001). BPD PRS was not associated with any gastrointestinal disorder. MDD PRS was positively associated with PUD (OR = 1.06, 95% CI: 1.04–1.07, *p* < 1E-16), GERD (OR = 1.07, 95% CI: 1.05–1.08, *p* < 1E-16), IBS (OR = 1.07, 95% CI: 1.05–1.09, *p* = 2E-11), and IBD (OR = 1.06, 95% CI: 1.02–1.10, *p* = 0.005; less than Bonferroni-corrected significance). OCD PRS was positively associated with PUD (OR = 1.02, 95% CI: 1.00–1.03, *p* = 0.01; less than Bonferroni-corrected significance), GERD (OR = 1.02, 95% CI: 1.01–1.03, *p* = 0.004; less than Bonferroni-corrected significance), and IBS (OR = 1.03, 95% CI: 1.01–1.05, *p* = 0.001). For the deciles of PRS with the lowest as a reference, the ORs between PRS for psychiatric and gastrointestinal disorders are plotted in Fig. 3 for visualization (detailed numbers in Supplementary Data 11).

**Fig. 4:**
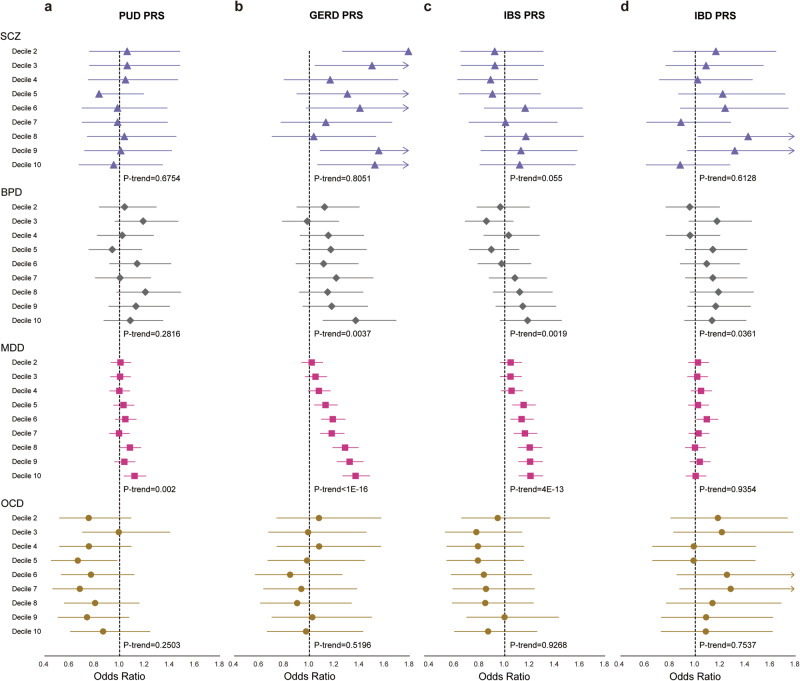
Association of decile of polygenic risk score (PRS) for gastrointestinal disorders with psychiatric disorders. **a** Peptic ulcer disease (PUD). **b** Gastroesophageal reflux disease (GERD). **c** Irritable bowel syndrome (IBS). **d** Inflammatory bowel disease (IBD). Odds ratio and 95% confidence interval for the highest nine deciles, respectively, compared to the lowest decile. Purple triangle indicates schizophrenia (SCZ), gray diamond indicates bipolar disorder (BPD), pink square indicates major depressive disorder (MDD), and brown circle indicates obsessive-compulsive disorder (OCD). Sample size = 106,796.

PUD PRS was associated with MDD (OR = 1.03, 95% CI: 1.01–1.05, *p* = 0.002). GERD PRS was associated with BPD (OR = 1.08, 95% CI: 1.03–1.14, *p* = 0.003) and MDD (OR = 1.11, 95% CI: 1.09–1.14, *p* < 1E-16). IBS PRS was associated with SCZ (OR = 1.08, 95% CI: 1.00–1.17, *p* = 0.04; less than Bonferroni-corrected significance), BPD (OR = 1.07, 95% CI: 1.02–1.12, *p* = 0.007; less than Bonferroni-corrected significance), and MDD (OR = 1.06, 95% CI: 1.05–1.08, *p* = 4E-13). IBD PRS was associated with BPD (OR = 1.05, 95% CI: 1.00–1.11, *p* = 0.03; less than Bonferroni-corrected significance). The ORs between deciles of PRS for gastrointestinal and psychiatric disorders are plotted in Fig. 4 (detailed numbers in Supplementary Data 12).

Adjusting several potential confounders, including body mass index (BMI), education attainment, and lifestyles (smoking, drinking, and exercise habit) did not substantively affect the PRS results (Supplementary Table 9). Further adjusting diet habit (including tea and coffee consumption, vegetarian, and late-night supper) also did not affect the results. After excluding individuals with a corresponding psychiatric/gastrointestinal disorder based on PRS, the results remained similar (Supplementary Table 10).

Modest sex differences, less than Bonferroni-corrected significance, were detected in PRS associations (Supplementary Table 11). SCZ PRS was associated with PUD in males but not in females. BPD PRS was associated with IBS in females but not in males. The effect of MDD PRS on IBS was greater in females than in males.

### Summary statistics-based genetic correlation

**Fig. 5:**
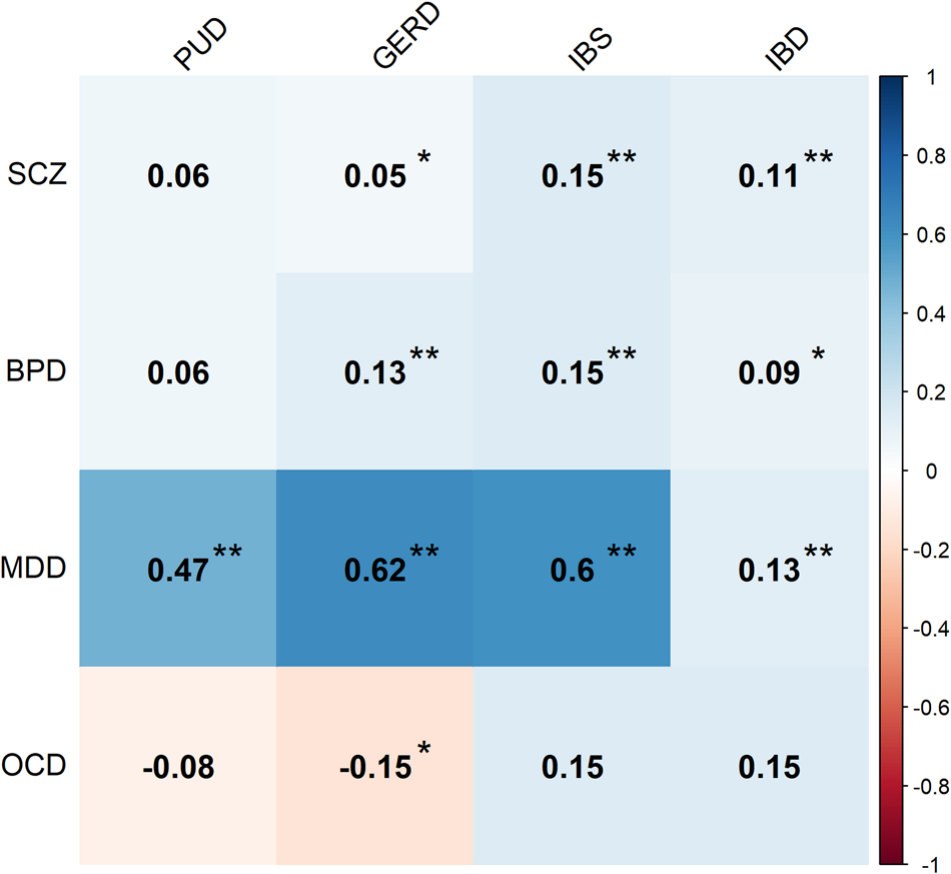
Genetic correlations between psychiatric disorders and gastrointestinal disorders. The value indicates the rg estimate. *indicates *p* < 0.05; **indicates *p* < 0.003125. schizophrenia (SCZ); bipolar disorder (BPD); major depressive disorder (MDD); obsessive-compulsive disorder (OCD); peptic ulcer disease (PUD); gastroesophageal reflux disease (GERD); irritable bowel syndrome (IBS); inflammatory bowel disease (IBD).

The genetic correlations between psychiatric and gastrointestinal disorders are shown in Fig. 5. There was a small positive genetic correlation (rg range, 0.11–0.15) between SCZ and IBS/IBD, BPD and GERD/IBS, and MDD and IBD. There was a modest positive genetic correlation (rg range, 0.47–0.62) between MDD and PUD/GERD/IBS.

### Mendelian randomization and causal association

Bidirectional causal associations (vertical pleiotropy) between psychiatric and gastrointestinal disorders using inverse variance weighted method are presented in Supplementary Fig 3. Genetically predicted MDD was associated with a higher risk of PUD, GERD, and IBS. Genetically predicted GERD and IBS was associated with a higher risk of MDD. Genetically predicted SCZ was associated with IBD. The results from alternative Mendelian randomization methods were generally consistent (Supplementary Data 13).

## Discussion

This study explored the underlying mechanisms of brain-gut comorbidity using three complementary approaches: testing comorbidity and familial coaggregation in different relatives, examining individual-level shared polygenetic loading, and evaluating the summary statistics-based genetic correlation and bidirectional causality between four psychiatric and four gastrointestinal disorders. Triangulating evidence from these analyses revealed familial coaggregation and a shared genetic etiology.

To our knowledge, this is the largest nationwide cohort study and one of the first studies to comprehensively explore shared genetic etiology underlying psychiatric and gastrointestinal comorbidity. Different complementary study designs were used in this study. Our findings of shared genetic etiology behind the brain-gut comorbidity in Taiwanese of East Asian populations replicated a recent study^46^ in European populations, which purely dependent on GWAS summary. Our study used large-scale individual health insurance data and individual genotyping data to perform comorbid, familial coaggregation and shared polygenic loading analyses in East Asian populations. Our nationwide cohort study has several unique characteristics, including no ascertainment bias because of the extensive coverage rate and large sample size (more than 4.5 million subjects). Our biobank study, with more than 130,000 community participants, used the latest GWAS discovery sample and state-of-the-art Bayesian method to optimize PRS prediction and polygenic association. Several potential confounders have been considered in the PRS association tests. Moreover, the disease information was identified with ICD diagnoses using the linkage to NHIRD rather than retrospective self-reporting that may lead to recall bias and result in misclassification and underestimation of the prevalence of diseases^67^.

Within-family analyses suggested that there was familial coaggregation between psychiatric and gastrointestinal disorders and implied a shared mechanism in brain-gut comorbidity. Although this could not rule out shared environmental contributions, the patterns of strength of association in different relatives and in varying numbers of affected relatives provided evidence for shared genetic etiology between psychiatric and gastrointestinal disorders. First, the association was greater in same-sex twins (mixing of monozygotic twins sharing ~100% of genes and dizygotic twins sharing ~50% of genes) than in full siblings (sharing ~50% of genes). Second, the association in full siblings was similar to that in either parent (all shared ~50% of the genes). Third, familial association was greater in two affected siblings than in one affected sibling. Fourth, familial association was greater in both affected parents than in either parent.

Gastrointestinal disorders are multifactorial and correlated with a shared etiological mechanism. *Helicobacter pylori* infection and pharmacotherapy with nonsteroidal anti-inflammatory drugs are major risk factors that lead to subsequent inflammation and mucosal damage^68^, although approximately 20% of PUD cases are not related to this^69^. This implies the importance of other pathogeneses, and the brain-to-gut pathway is an emerging one. In addition, individuals with higher genetic liability for MDD are at a higher risk for PUD and GERD^26^. This study provided extensive evidence of the influence of genetic liability for a wide range of psychiatric disorders on PUD, GERD, IBS, and IBD.

Our findings that the polygenic association between MDD and gastrointestinal disorders are in line with the genetic correlation between them. The PRS for GERD/IBS/IBD was associated with BPD, which was consistent with the observed genetic correlation between them; however, BPD PRS was not associated with GERD/IBS/IBD. Both PRS analyses and genetic correlation analyses showed no genetic overlap between BPD and PUD, which implied the role of environmental factors in comorbidity and familial coaggregation between them. Our finding that SCZ PRS was associated with IBS, and vice versa, echoed the genetic correlation between SCZ and IBS. Additionally, our results that higher OCD PRS increased the risk for PUD, GERD, and IBS in Taiwanese samples of East Asian populations are novel and important for the understanding of brain-gut comorbidity; the opposite direction for the summary statistics-based genetic correlation between OCD and GERD (not passed Bonferroni correction significance) in European populations may be due to different populations.

The interplay between psychiatric and gastrointestinal disorders implies the importance of developing screening and treatment strategies^70^. Our analysis of within-individual and within-family association between psychiatric and gastrointestinal disorders suggested that individual and familial psychological factors are important in the development of gastrointestinal disorders, and individuals with psychiatric disorders or with psychiatric disorder-affected relatives should be monitored and screened for gastrointestinal disorders. Psychiatric interventions, such as antidepressants and psychological therapies were effective in the treatment of gastrointestinal disorders^16,17,71^. Conversely, the effect of treatment for gastrointestinal disorders on psychiatric comorbidity was summarized^71^.

Psychological stress induces systemic and mucosal proinflammatory responses^72^, increases small intestinal permeability^73^, and triggers gastrointestinal symptoms and disorders^74,75^. Vice versa, gut microbial dysbiosis influences brain chemistry and contributes to psychiatric disorder^76–78^. Our PRS analyses further suggested that pleiotropy contributed to brain-gut comorbidity, and the association of psychiatric PRS with a gastrointestinal disorder might be attributed to psychological suffering in individuals with a higher genetic load for psychiatric disorders. In our within-family analyses, parental SCZ was associated with PUD/GERD and parental BPD/MDD was associated with IBD; however, reversely, these trans-generational associations were not observed. This implied that parental psychiatric disorders could create a stressful family environment that affects gut health. Adequate prevention and treatment of psychological distress may be crucial for gut health.

Psychological stress triggers and exacerbates immune-mediated symptoms and diseases^79^. By contrast, immune processes are involved in the development of psychiatric disorders^80^. GWAS have identified immune loci and multiple immune signaling pathways enriched for psychiatric risk^47,81^. Shared genetic loadings between psychiatric disorders and immune-mediated diseases, such as IBD^23,27,30^, suggested the role of immune dysfunction in the central nervous system.

Sex differences in psychiatric/gastrointestinal disorders have been documented^82,83^, but sex differences in brain-gut comorbidity have not been adequately explored. The within-family association of family history of MDD in full siblings with PUD risk was larger in females. In addition, we observed that the effect of PRS for BPD and MDD on IBS was greater in females. A GWAS on IBS has also identified female-specific susceptibility loci^42^, which have been linked to sex-specific traits and sex hormones. Sex hormones have an influence on gastrointestinal functions^83–85^. Sex hormones and other hormones could contribute to regulatory signals along the axis of brain-gut interactions and influence gut motor function and mucosal immune activation^84,86^.

This study has some limitations. First, the cohort study used NHIRD between 1998 and 2020 to follow-up the study samples and identify their diseases; only individuals, including the study samples and their relatives, who were alive during the study period, were included. Disease diagnoses were missed if they did not use any medical services during the catchment period. Second, given the uncertainty in establishing the exact disease onset time using national insurance claims (the onset can have left-censoring if it was earlier than the study start year of 1997) and the extensiveness of diagnostic ascertainment (99% of the coverage), the cumulative incidence is a more stable metric than instantaneous incidence; hence, we decided to perform logistic regression, which is robust to the characteristics of our data, rather than the Cox model. Future studies should ascertain the onset time and estimate the risk trajectories and order between the comorbidity of psychiatric and gastrointestinal disorders. Third, the sample size for both parents were affected (P+M+) and same-sex twins was relatively limited, and it resulted in wider confidence interval estimates. Fourth, genetic architecture differs across populations; hence, using cross-ancestry GWAS results to derive PRS could lower predictive variance^87^. The discovery sample used in this study was mainly of European ancestry, and we applied the PRS-CS/PRS-CSx to improve cross-population polygenic prediction in Asian samples. In addition, PRS only considers common variants and thus captures only limited heritability estimated from family studies. Fifth, the recruited biobank participants were not representative of the population; hence, selection bias for healthy volunteers was possible. Nevertheless, a valid assessment of the genetic associations may be generalizable. Sixth, medications and microbiomes were not considered in this study, and their role, as confounders, mediators, modifiers, or prognostic factors, in comorbidity and shared genetic architecture between psychiatric disorders and gastrointestinal disorders should be further studied. Finally, we only included individuals of Taiwanese ancestry in family-based and PRS analyses. Therefore, further investigations are warranted to determine whether our findings can be generalized to other populations.

In conclusion, our population-based cohort and biobank study showed that there was familial coaggregation and shared genetic etiology behind the co-occurrence of psychiatric and gastrointestinal disorders. Thus, efforts to elucidate the mechanisms of brain-gut comorbidity have implications for prevention and therapeutic planning.

## Supplementary information

Peer Review File

Supplementary Information

Description of Additional Supplementary Files

Supplementary data

Reporting Summary

## Data Availability

Publicly available data are available from the following sites: GWAS summary results for psychiatric diseases were downloaded from https://www.med.unc.edu/pgc/; GWAS summary results for PUD were downloaded from https://cnsgenomics.com/content/data; GWAS summary results for GERD were downloaded from http://ftp.ebi.ac.uk/pub/databases/gwas/summary_statistics/GCST90000001-GCST90001000/GCST90000514/; GWAS summary results for IBS were downloaded from http://ftp.ebi.ac.uk/pub/databases/gwas/summary_statistics/GCST90016001-GCST90017000/GCST90016564/; GWAS summary results for IBD were downloaded from http://ftp.ebi.ac.uk/pub/databases/gwas/summary_statistics/GCST004001-GCST005000/GCST004131/. The NHIRD used in this study is held by the Taiwan Ministry of Health and Welfare and under controlled access. Researchers interested in accessing the data set can submit an application form to the Ministry of Health and Welfare requesting access. Taiwan Biobank data used in this study is under controlled access. Application to access can be made to the Taiwan Biobank. All other data are available from the corresponding author on reasonable request. The source data for Fig. 1 is in Supplementary Table 4 and Supplementary Data 1–4. The source data for Fig. 2 is in Supplementary Table 5 and Supplementary Data 5–8. The source data for Fig. 3 is in Supplementary Data 11. The source data for Fig. 4 is in Supplementary Data 12.

## Code availability

The code used in this study is available from the following websites: PRS-CSx: https://github.com/getian107/PRScsx (10.1038/s41588-022-01054-7)^56^; PRS-CS: https://github.com/getian107/PRScs (10.1038/s41467-019-09718-5)^57^; LD score regression: https://github.com/bulik/ldsc (10.1038/ng.3211 and 10.1038/ng.3406)^60,61^.
