# Supplementary material for: A population-based study of familial coaggregation and shared genetic etiology of psychiatric and gastrointestinal disorders
Source: Commun Med (Lond). 2024 Sep 19;4:180. doi: 10.1038/s43856-024-00607-7 (PMC11413006; doi:10.1038/s43856-024-00607-7)
Supplement: Supplementary file 2 — Supplementary Information [file 43856_2024_607_MOESM2_ESM.pdf]

Supplementary Figures and Tables

Supplementary Figure 1. Sex-stratified association of individual history and family history, including parents, same-sex twins, and full siblings, of psychiatric disorders with the risk of gastrointestinal disorders. Blue indicates males; red indicated females

Supplementary Figure 2. Sex-stratified association of individual history and family history, including parents, same-sex twins, and full siblings, of gastrointestinal disorders with the risk of psychiatric disorders. Blue indicates males; red indicated female

Supplementary Figure 3. Bidirectional causality estimates between psychiatric and gastrointestinal disorders using IVW method.

Supplementary Table 1. ICD code for psychiatric disorders and gastrointestinal disorders

Supplementary Table 2. Distribution of demographic characteristics, psychiatric disorders, and gastrointestinal disorders among the study cohorts

Supplementary Table 3. Sex-stratified prevalence of the psychiatric disorders, and gastrointestinal disorders in total cohort (4,504,612 individuals)

Supplementary Table 4. Distributions and associations of the comorbidities of psychiatric disorders and gastrointestinal disorders (sample size=4,504,612)

Supplementary Table 5. Distributions and associations of the comorbidities of psychiatric disorders and gastrointestinal disorders (sample size=4,504,612)

Supplementary Table 6. Distribution of the demographics, psychiatric disorders, and gastrointestinal disorders in 106796 unrelated participants from the Taiwan Biobank

Supplementary Table 7. The association of polygenic risk score for psychiatric disorders and gastrointestinal disorders and their corresponding diagnosis in Taiwan Biobank (n=106,976)

Supplementary Table 8. Association of the polygenic risk score for psychiatric disorders with gastrointestinal disorders and gastrointestinal disorders with psychiatric disorders (n=106,796)

Supplementary Table 9. Association of the polygenic risk score for psychiatric disorders with gastrointestinal disorders and gastrointestinal disorders with psychiatric disorders after adjusting potential confounding factors (n=106,796)

Supplementary Table 10. Association of the polygenic risk score for psychiatric disorders (excluding individuals with a corresponding psychiatric disorder) with gastrointestinal disorders. A total of 106,127, 105,071, 92,828, and 106,276 individuals remained for PRS testing for SCZ, BPD, MDD, and OCD, respectively. Association of the polygenic risk score for gastrointestinal disorders (excluding individuals with a corresponding gastrointestinal disorder) with psychiatric disorders. A total of 61,571, 74,104, 91,739, and 104,151 individuals remained for PRS testing for PUD, GERD, IBS, and IBD, respectively

Supplementary Table 11. Sex differences in the association of polygenic risk score for psychiatric disorders with gastrointestinal disorders (n=106,796)

Supplementary Figure 1. Sex-stratified association of individual history and family history, including parents, same-sex twins, and full siblings, of psychiatric disorders with the risk of gastrointestinal disorders. Blue indicates males; red indicated females

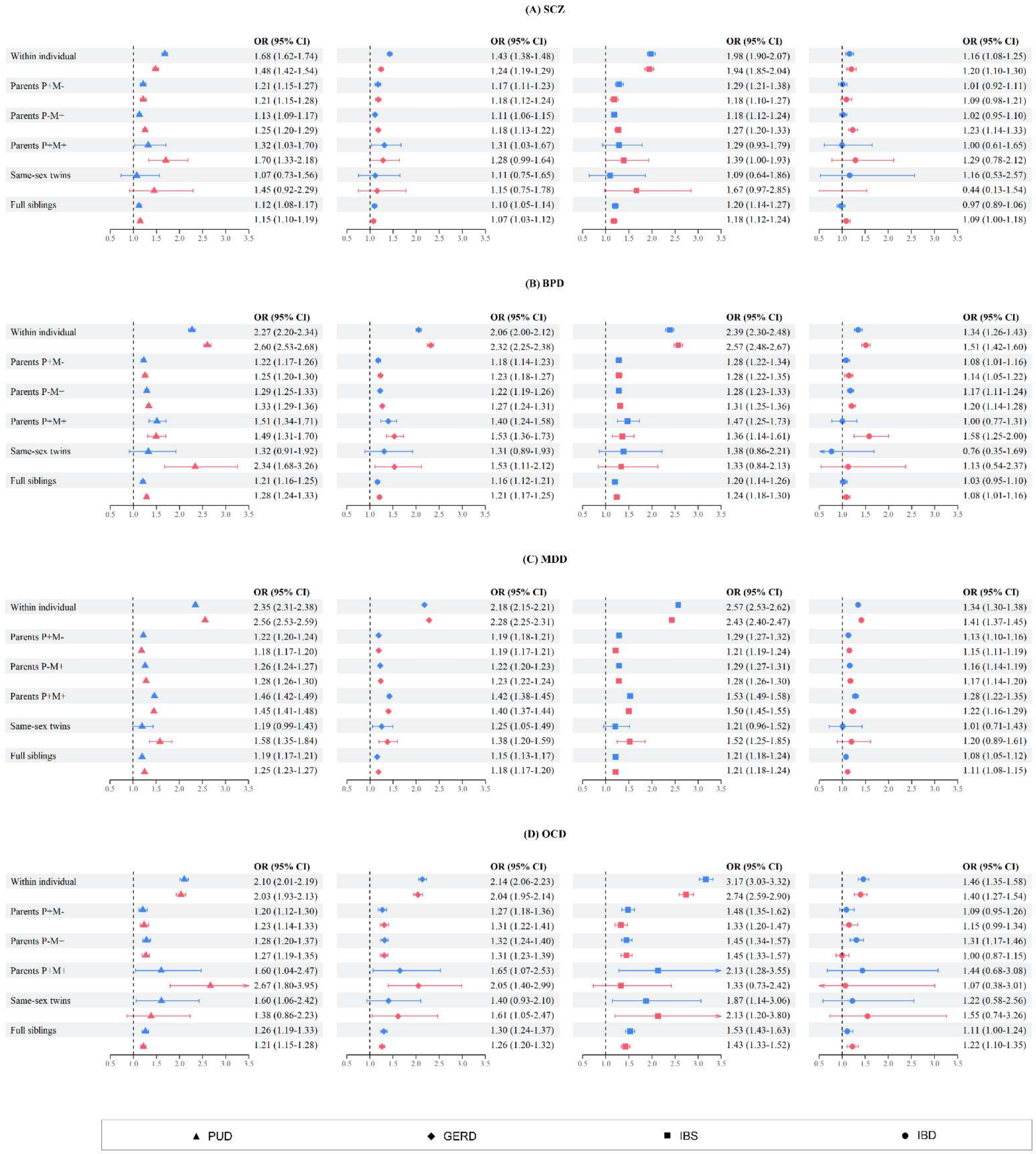

Supplementary Figure 2. Sex-stratified association of individual history and family history, including parents, same-sex twins, and full siblings, of gastrointestinal disorders with the risk of psychiatric disorders. Blue indicates males; red indicated female

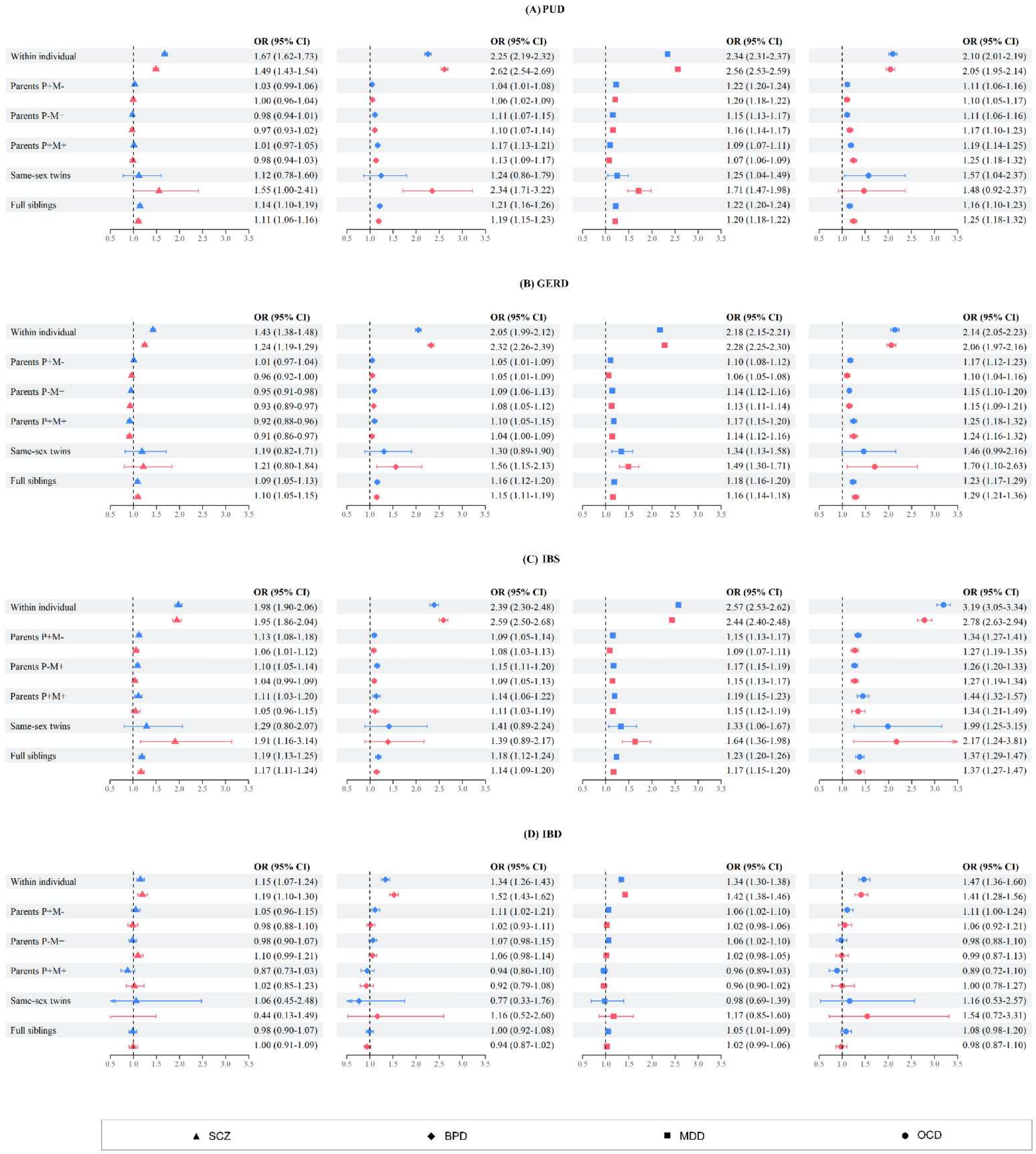

Supplementary Figure 3. Bidirectional causality estimates between psychiatric and gastrointestinal disorders using IVW method.

Exposure	Outcome	Odds Ratio	LCL	UCL	Forest Plot	P-value
SCZ	PUD	1.03	0.99	1.07		7E-02
	GERD	1.03	1.01	1.05		9E-02
	IBS	1.03	1.01	1.05		1E-02
	IBD	1.15	1.11	1.20		2E-12
BPD	PUD	0.97	0.92	1.03		4E-01
	GERD	1.00	0.94	1.06		9E-01
	IBS	1.04	0.98	1.10		2E-01
	IBD	1.06	0.96	1.17		2E-01
MDD	PUD	1.28	1.12	1.47		2E-04
	GERD	2.01	1.83	2.22		<1E-16
	IBS	1.54	1.39	1.70		<1E-16
	IBD	1.23	0.98	1.56		8E-02
OCD	PUD	0.99	0.95	1.03		6E-01
	GERD	0.98	0.96	1.00		9E-02
	IBS	1.00	0.96	1.04		9E-01
	IBD	1.04	0.98	1.10		1E-01
PUD	SCZ	1.02	0.86	1.22		8E-01
	BPD	0.98	0.87	1.10		8E-01
	MDD	0.98	0.94	1.02		3E-01
	OCD	0.97	0.62	1.52		9E-01
GERD	SCZ	1.16	0.97	1.39		9E-02
	BPD	1.14	0.97	1.33		8E-02
	MDD	1.62	1.52	1.71		<1E-16
	OCD	1.05	0.78	1.41		7E-01
IBS	SCZ	1.12	0.77	1.62		5E-01
	BPD	1.07	0.64	1.79		8E-01
	MDD	1.32	1.11	1.58		1E-03
	OCD	1.40	0.67	2.96		4E-01
IBD	SCZ	1.00	0.98	1.02		7E-01
	BPD	0.99	0.97	1.01		5E-01
	MDD	1.00	1.00	1.00		9E-01
	OCD	1.02	0.96	1.08		5E-01

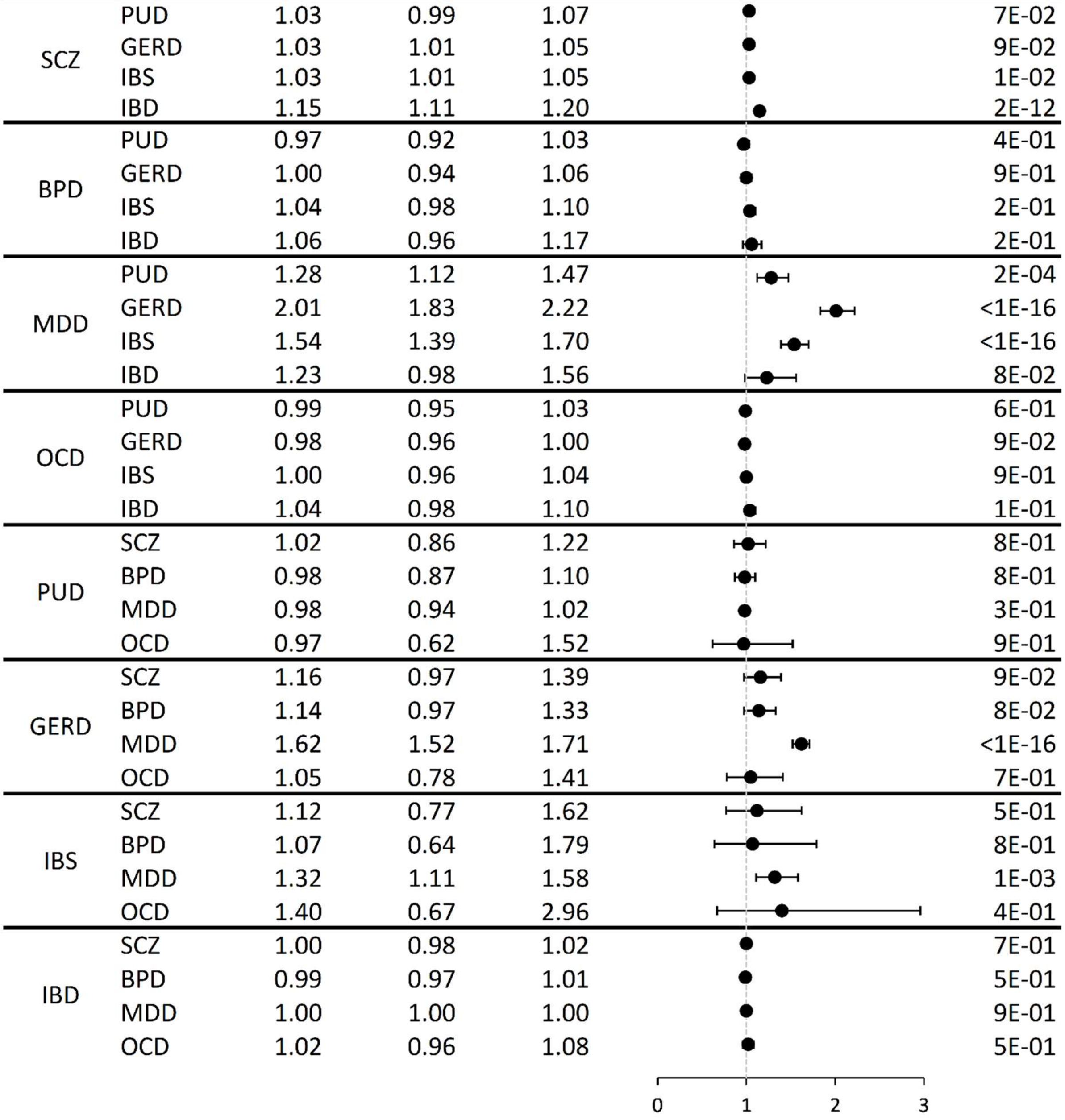

Supplementary Table 1. ICD code for psychiatric disorders and gastrointestinal disorders.

Diseases	ICD 9	ICD 10
Schizophrenia	295.x	F20, F25
Bipolar disorder	296.0-296.1, 296.4-296.8	F30, F31, F34.0,
Major depressive disorder	296.2x, 296.3x, 300.4, 311	F32, F33, F34.1
Obsessive-compulsive disorder	300.3	F42
Peptic Ulcer Disease	531-534	K25-K28
Gastroesophageal reflux disease	530.11, 530.81	K21
Irritable Bowel Syndrome	564.1	K58
Inflammatory bowel disease	555, 556	K50, K51

Supplementary Table 2. Distribution of demographic characteristics, psychiatric disorders, and gastrointestinal disorders among the study cohorts

	Total cohort N=4,504,612		Same-sex twins N=51,664		Full-sibling N=3,322,959	
	n	%	n	%	n	%
Gender						
Female	2121632	47.1	24656	47.7	1605811	48.3
Male	2382980	52.9	27008	52.3	1717148	51.7
Age, mean (SD)	32.7	7.6	29.8	6.4	32.3	6.8
Birth Cohort						
1970-1979	730779	16.2	3066	5.9	389485	11.7
1980-1989	1713660	38.0	16326	31.6	1376511	41.4
1990-1999	2060173	45.7	32272	62.5	1556963	46.9
Income level						
<33300	1524213	33.8	18225	35.3	1108876	33.4
33300-50600	1495586	33.2	16550	32.0	1113142	33.5
>50600	1484813	33.0	16889	32.7	1100941	33.1
Urbanization						
Urban	2460402	54.6	28538	55.2	1774321	53.4
Sub-urban	1676293	37.2	18792	36.4	1264613	38.1
Rural	367917	8.2	4334	8.4	284025	8.6
PUD	565860	12.6	4823	9.3	400914	12.1
GERD	591976	13.1	5688	11.0	430236	13.0
IBS	262960	5.8	2547	4.9	188724	5.7
IBD	141337	3.1	1691	3.3	101379	3.1
SCZ	38447	0.9	420	0.8	27348	0.8
BPD	49325	1.1	488	0.9	35613	1.1
MDD	272839	6.1	2597	5.0	195426	5.9
OCD	23810	0.5	341	0.7	17083	0.5

Supplementary Table 3. Sex-stratified prevalence of the psychiatric disorders, and gastrointestinal disorders in total cohort (4,504,612 individuals).

Variable	Female N=2121632 n (%)	Male N=2382980 n (%)	p-value*
Psychiatric disorders			
SCZ	16167 (0.76)	22280 (0.93)	<1E-16
BPD	24980 (1.17)	24435 (1.03)	<1E-16
MDD	153649 (7.24)	119190 (5.00)	<1E-16
OCD	9953 (0.47)	13857 (0.58)	<1E-16
Gastrointestinal disorders			
PUD	280960 (13.24)	284900 (11.96)	<1E-16
GERD	303412 (14.30)	288564 (12.11)	<1E-16
IBS	126158 (5.95)	136802 (5.74)	<1E-16
IBD	64518 (3.04)	76819 (3.22)	<1E-16

*p-value for testing sex difference; Chi-Square test for categorical variables.

Supplementary Table 4. Distributions and associations of the comorbidities of psychiatric disorders and gastrointestinal disorders (sample size=4,504,612).

		N	PUD						GERD						IBS						IBD					
			n	%	aOR	LCL	UCL	p-value	n	%	aOR	LCL	UCL	p-value	n	%	aOR	LCL	UCL	p-value	n	%	aOR	LCL	UCL	p-value
SCZ	No	4466165	557281	12.48	1.00	-	-	-	584770	13.09	1.00	-	-	-	258357	5.78	1.00	-	-	-	140024	3.14	1.00	-	-	-
	Yes	38447	8579	22.31	1.59	1.55	1.63	<1E-16	7206	18.74	1.34	1.30	1.37	<1E-16	4603	11.97	1.95	1.89	2.01	<1E-16	1313	3.42	1.17	1.10	1.23	4E-08
BPD	No	4455287	552308	12.40	1.00	-	-	-	579138	13.00	1.00	-	-	-	256080	5.75	1.00	-	-	-	139259	3.13	1.00	-	-	-
	Yes	49325	13552	27.53	2.42	2.37	2.47	<1E-16	12838	26.07	2.18	2.14	2.23	<1E-16	6880	13.97	2.47	2.40	2.53	<1E-16	2078	4.22	1.42	1.36	1.48	<1E-16
MDD	No	4231773	492358	11.63	1.00	-	-	-	521558	12.32	1.00	-	-	-	226664	5.36	1.00	-	-	-	130532	3.08	1.00	-	-	-
	Yes	272839	73502	26.94	2.45	2.43	2.47	<1E-16	70418	25.81	2.23	2.21	2.25	<1E-16	36296	13.30	2.48	2.45	2.51	<1E-16	10805	3.96	1.36	1.34	1.39	<1E-16
OCD	No	4480802	560456	12.51	1.00	-	-	-	586242	13.08	1.00	-	-	-	259192	5.78	1.00	-	-	-	140292	3.13	1.00	-	-	-
	Yes	23810	5404	22.70	2.05	1.99	2.12	<1E-16	5734	24.08	2.08	2.02	2.15	<1E-16	3768	15.83	2.95	2.85	3.06	<1E-16	1045	4.39	1.41	1.32	1.50	<1E-16

aOR: adjusted sex, birth cohort, age, income level, and urbanization level

Supplementary Table 5. Distributions and associations of the comorbidities of psychiatric disorders and gastrointestinal disorders (sample size=4,504,612).

		N	SCZ						BPD						MDD						OCD					
			n	%	aOR	LCL	UCL	p-value	n	%	aOR	LCL	UCL	p-value	n	%	aOR	LCL	UCL	p-value	n	%	aOR	LCL	UCL	p-value
PUD	No	3938752	29868	0.76	1.00	-	-	-	35773	0.91	1.00	-	-	-	199337	5.06	1.00	-	-	-	18406	0.47	1.00	-	-	-
	Yes	565860	8579	1.52	1.59	1.55	1.63	<1E-16	13552	2.39	2.43	2.38	2.48	<1E-16	73502	12.99	2.45	2.43	2.48	<1E-16	5404	0.96	2.07	2.01	2.14	<1E-16
GERD	No	3912636	31241	0.80	1.00	-	-	-	36487	0.93	1.00	-	-	-	202421	5.17	1.00	-	-	-	18076	0.46	1.00	-	-	-
	Yes	591976	7206	1.22	1.34	1.30	1.37	<1E-16	12838	2.17	2.19	2.14	2.23	<1E-16	70418	11.90	2.23	2.21	2.25	<1E-16	5734	0.97	2.10	2.03	2.16	<1E-16
IBS	No	4241652	33844	0.80	1.00	-	-	-	42445	1.00	1.00	-	-	-	236543	5.58	1.00	-	-	-	20043	0.47	1.00	-	-	-
	Yes	262960	4603	1.75	1.96	1.90	2.02	<1E-16	6880	2.62	2.48	2.42	2.55	<1E-16	36296	13.80	2.49	2.46	2.52	<1E-16	3768	1.43	3.01	2.90	3.11	<1E-16
IBD	No	4363275	37134	0.85	1.00	-	-	-	47247	1.08	1.00	-	-	-	262034	6.01	1.00	-	-	-	22765	0.52	1.00	-	-	-
	Yes	141337	1313	0.93	1.17	1.10	1.23	4E-08	2078	1.47	1.43	1.37	1.49	<1E-16	10805	7.64	1.38	1.35	1.41	<1E-16	1045	0.74	1.45	1.36	1.54	<1E-16

aOR: adjusted sex, birth cohort, age, income level, and urbanization level

Supplementary Table 6. Distribution of the demographics, psychiatric disorders, gastrointestinal disorders, BMI, education attainment, diet, and lifestyle in 106796 unrelated participants from the Taiwan Biobank.

Variable	Female N=67882 n (%)	Male N=38914 n (%)	p-value*
Age			<1E-16
30-39	14498 (21.36)	8956 (23.01)	
40-49	17299 (25.48)	9842 (25.29)	
50-59	21995 (32.40)	10721 (27.55)	
60-70	14090 (20.76)	9395 (24.14)	
Psychiatric disorders			
SCZ	393 (0.58)	276 (0.71)	9E-03
BPD	1205 (1.78)	520 (1.34)	4E-08
MDD	10234 (15.08)	3734 (9.60)	<1E-16
OCD	297 (0.44)	223 (0.57)	2E-03
Gastrointestinal disorders			
PUD	29415 (43.33)	15810 40.63)	<1E-16
GERD	21592 (31.81)	11100 (28.52)	<1E-16
IBS	9744 (14.35)	5313 (13.65)	2E-03
IBD	1668 (2.46)	977 (2.51)	6E-01
BMI[a]	23.59 (3.77)	25.42 (3.55)	
Education attainment[b]			<1E-16
Elementary school and below	3910 (5.76)	1001 (2.57)	
Junior high school	5534 (8.15)	2098 (5.39)	
Senior high/Vocational school	21649 (31.89)	9392 (24.14)	
University/College	30930 (45.56)	20245 (52.02)	
Master and above	5838 (8.60)	6170 (15.86)	
Diet			
Tea[c]	3497 (5.15)	2798 (7.19)	<1E-16
Coffee[c]	4516 (6.65)	2249 (5.78)	2E-08
Vegetarian[d]	1300 (1.92)	522 (1.34)	3E-12
Late-night supper[d]	3206 (4.72)	2376 (6.11)	<1E-16
Lifestyle			
Tobacco smoking[e]			<1E-16
Never	60606 (89.28)	16563 (42.56)	
Ever	1821 (2.68)	9194 (23.63)	
Current	2059 (3.03)	8012 (20.59)	
Alcohol drinking[f]			<1E-16
Never	65895 (97.07)	31536 (81.04)	
Ever	616 (0.91)	2219 (5.70)	
Current	1314 (1.94)	5123 (13.16)	
Exercise habit[g]	26553 (39.12)	16321 (41.94)	<1E-16

missing data: a= 70; b=29; c=88145; d=88146; e=8541; f=93; g=57

Supplementary Table 7. The association of polygenic risk score for psychiatric disorders and gastrointestinal disorders and their corresponding diagnosis in Taiwan Biobank (n=106,976).

PRS	Method	Outcome	aOR	p-value	ΔR^2(%)
SCZ PRS	PRS-CSx	SCZ	1.64	<1E-16	2.05
SCZ PRS	PRS-CS	SCZ	1.62	<1E-16	1.98
BPD PRS	PRS-CS	BPD	1.19	8E-13	0.31
MDD PRS	PRS-CSx	MDD	1.18	<1E-16	0.55
MDD PRS	PRS-CS	MDD	1.18	<1E-16	0.54
OCD PRS	PRS-CS	OCD	1.08	1E-01	0.04
PUD PRS	PRS-CS	PUD	1.04	9E-10	0.04
GERD PRS	PRS-CS	GERD	1.09	<1E-16	0.23
IBS PRS	PRS-CS	IBS	1.06	6E-11	0.08
IBD PRS	PRS-CS	IBD	1.02	2E-01	0.01

aOR is estimated from logistic regression models with adjustment for age, sex, batch effect, and 20 PCs.
ΔR^2(%): increased in Nagelkerke pseudo R^2 when adding the PRS into the model including age, sex, batch effect, and 20 PCs.

Supplementary Table 8. Association of the polygenic risk score for psychiatric disorders with gastrointestinal disorders and gastrointestinal disorders with psychiatric disorders (n=106,796).

	PUD		GERD		IBS		IBD	
	aOR (95% CI)	p-value	aOR (95% CI)	p-value	aOR (95% CI)	p-value	aOR (95% CI)	p-value
SCZ PRS	1.00 (0.99-1.01)	8E-01	1.00 (0.99-1.01)	9E-01	1.03 (1.01-1.05)	1E-03	0.98 (0.94-1.02)	3E-01
BPD PRS	1.01 (1.00-1.02)	2E-01	1.00 (0.99-1.02)	7E-01	1.01 (1.00-1.03)	1E-01	1.01 (0.97-1.05)	8E-01
MDD PRS	1.06 (1.04-1.07)	9E-16	1.07 (1.05-1.08)	<1E-16	1.07 (1.05-1.09)	1E-12	1.06 (1.02-1.10)	5E-03
OCD PRS	1.02 (1.00-1.03)	1E-02	1.02 (1.01-1.03)	4E-03	1.03 (1.01-1.05)	1E-03	1.01 (0.97-1.05)	6E-01
	SCZ		BPD		MDD		OCD	
	aOR (95% CI)	p-value	aOR (95% CI)	p-value	aOR (95% CI)	p-value	aOR (95% CI)	p-value
PUD PRS	0.98 (0.91-1.06)	7E-01	1.03 (0.98-1.08)	3E-01	1.03 (1.01-1.05)	2E-03	0.96 (0.88-1.05)	4E-01
GERD PRS	1.03 (0.95-1.11)	5E-01	1.08 (1.03-1.14)	3E-03	1.11 (1.09-1.14)	<1E-16	0.98 (0.90-1.07)	7E-01
IBS PRS	1.08 (1.00-1.17)	4E-02	1.07 (1.02-1.12)	7E-03	1.06 (1.05-1.08)	4E-16	0.99 (0.90-1.08)	8E-01
IBD PRS	1.02 (0.94-1.10)	6E-01	1.05 (1.00-1.11)	3E-02	1.00 (0.98-1.02)	1E+00	1.01 (0.93-1.10)	8E-01

aOR: adjusted odds ratio with adjustment for sex, age, batch version, and 20 population stratification dimensions.

Supplementary Table 9. Association of the polygenic risk score for psychiatric disorders with gastrointestinal disorders and gastrointestinal disorders with psychiatric disorders after adjusting potential confounding factors (n=106,796).

	PUD		GERD		IBS		IBD	
	aOR (95% CI)	p-value	aOR (95% CI)	p-value	aOR (95% CI)	p-value	aOR (95% CI)	p-value
M1								
SCZ PRS	1.00 (0.99-1.01)	9E-01	1.00 (0.99-1.02)	8E-01	1.03 (1.01-1.04)	4E-03	0.98 (0.94-1.02)	3E-01
BPD PRS	1.01 (1.00-1.02)	2E-01	1.00 (0.99-1.01)	9E-01	1.01 (0.99-1.03)	3E-01	1.00 (0.96-1.04)	1E+00
MDD PRS	1.04 (1.03-1.05)	1E-15	1.06 (1.04-1.07)	9E-16	1.05 (1.03-1.08)	5E-05	1.06 (1.02-1.11)	5E-03
OCD PRS	1.02 (1.01-1.03)	5E-03	1.02 (1.01-1.03)	3E-03	1.03 (1.01-1.05)	2E-03	1.01 (0.97-1.05)	6E-01
M2								
SCZ PRS	1.00 (0.99-1.01)	8E-01	1.00 (0.99-1.02)	9E-01	1.03 (1.01-1.04)	5E-03	0.98 (0.94-1.02)	3E-01
BPD PRS	1.01 (1.00-1.02)	2E-01	1.00 (0.99-1.01)	1E+00	1.01 (0.99-1.03)	3E-01	1.00 (0.96-1.04)	1E+00
MDD PRS	1.04 (1.03-1.06)	9E-08	1.06 (1.04-1.07)	9E-16	1.05 (1.03-1.08)	5E-05	1.06 (1.02-1.11)	5E-03
OCD PRS	1.02 (1.00-1.03)	8E-03	1.02 (1.01-1.03)	4E-03	1.03 (1.01-1.05)	2E-03	1.01 (0.97-1.05)	6E-01
	SCZ		BPD		MDD		OCD	
M1								
PUD PRS	1.00 (0.90-1.12)	1E+00	1.03 (0.96-1.12)	4E-01	1.03 (1.00-1.06)	4E-02	0.92 (0.81-1.04)	2E-01
GERD PRS	0.97 (0.88-1.07)	6E-01	1.04 (0.98-1.12)	2E-01	1.09 (1.07-1.12)	1E-13	0.98 (0.87-1.10)	7E-01
IBS PRS	1.09 (1.00-1.19)	5E-02	1.05 (0.99-1.11)	1E-01	1.05 (1.03-1.08)	5E-05	0.97 (0.88-1.08)	6E-01
IBD PRS	1.02 (0.95-1.11)	6E-01	1.06 (1.01-1.12)	2E-02	1.00 (0.98-1.02)	9E-01	1.01 (0.92-1.10)	9E-01
M2								
PUD PRS	1.00 (0.90-1.12)	9E-01	1.04 (0.96-1.12)	4E-01	1.03 (1.00-1.06)	4E-02	0.92 (0.81-1.04)	2E-01
GERD PRS	0.97 (0.88-1.07)	6E-01	1.04 (0.98-1.11)	2E-01	1.09 (1.07-1.12)	1E-13	0.98 (0.87-1.10)	7E-01
IBS PRS	1.09 (1.00-1.19)	5E-02	1.05 (0.99-1.11)	1E-01	1.05 (1.03-1.08)	5E-05	0.97 (0.88-1.08)	6E-01
IBD PRS	1.02 (0.95-1.11)	6E-01	1.06 (1.01-1.12)	2E-02	1.00 (0.98-1.02)	9E-01	1.01 (0.92-1.10)	9E-01

M1: adjusted odds ratio with adjustment for sex, age, batch version, 20 population stratification dimensions, BMI, education attainment, and lifestyle.

M2: adjusted odds ratio with adjustment for sex, age, batch version, 20 population stratification dimensions, BMI, education attainment, diet, and lifestyle.

Supplementary Table 10. Association of the polygenic risk score for psychiatric disorders (excluding individuals with a corresponding psychiatric disorder) with gastrointestinal disorders. A total of 106,127, 105,071, 92,828, and 106,276 individuals remained for PRS testing for SCZ, BPD, MDD, and OCD, respectively.Association of the polygenic risk score for gastrointestinal disorders (excluding individuals with a corresponding gastrointestinal disorder) with psychiatric disorders. A total of 61,571, 74,104, 91,739, and 104,151 individuals remained for PRS testing for PUD, GERD, IBS, and IBD, respectively.

	PUD		GERD		IBS		IBD	
	aOR (95% CI)	p-value	aOR (95% CI)	p-value	aOR (95% CI)	p-value	aOR (95% CI)	p-value
SCZ PRS	1.00 (0.98-1.01)	6E-01	1.00 (0.99-1.01)	1E+00	1.03 (1.01-1.05)	3E-03	0.98 (0.94-1.02)	3E-01
BPD PRS	1.01 (1.00-1.02)	3E-01	1.00 (0.99-1.01)	1E+00	1.01 (0.99-1.03)	3E-01	1.00 (0.96-1.04)	9E-01
MDD PRS	1.04 (1.03-1.05)	1E-15	1.06 (1.04-1.07)	9E-16	1.05 (1.03-1.07)	5E-07	1.06 (1.02-1.11)	6E-03
OCD PRS	1.02 (1.00-1.03)	1E-02	1.02 (1.01-1.03)	5E-03	1.03 (1.01-1.05)	1E-03	1.01 (0.97-1.05)	7E-01
	SCZ		BPD		MDD		OCD	
	aOR (95% CI)	p-value	aOR (95% CI)	p-value	aOR (95% CI)	p-value	aOR (95% CI)	p-value
PUD PRS	1.03 (0.92-1.14)	6E-01	1.05 (0.97-1.13)	3E-01	1.03 (1.01-1.06)	2E-02	0.91 (0.80-1.04)	2E-01
GERD PRS	1.04 (0.95-1.14)	4E-01	1.08 (1.01-1.16)	2E-02	1.11 (1.08-1.13)	<1E-16	0.97 (0.87-1.09)	7E-01
IBS PRS	1.10 (1.01-1.20)	3E-02	1.06 (1.00-1.12)	6E-02	1.06 (1.04-1.08)	1E-09	0.97 (0.88-1.08)	6E-01
IBD PRS	1.02 (0.95-1.11)	6E-01	1.06 (1.01-1.12)	2E-02	1.00 (0.98-1.02)	1E+00	1.01 (0.92-1.10)	9E-01

aOR: adjusted odds ratio with adjustment for sex, age, batch version, and 20 population stratification dimensions.

Supplementary Table 11. Sex differences in the association of polygenic risk score for psychiatric disorders with gastrointestinal disorders (n=106,796).

	Male		Female		Sex-PRS interaction
	aOR (95% CI)	p-value	aOR (95% CI)	p-value	p-value
SCZ PRS with PUD	1.02 (1.00-1.04)	5E-02	0.99 (0.97-1.00)	7E-02	1E-02
BPD PRS with IBS	0.99 (0.96-1.02)	4E-01	1.03 (1.01-1.05)	1E-02	2E-02
MDD PRS with IBS	1.04 (1.01-1.08)	4E-03	1.08 (1.06-1.11)	6E-11	4E-02

aOR: adjusted odds ratio with adjustment for sex, age, batch version, and 20 population stratification dimensions
