# Supplementary material for: A population-based study of familial coaggregation and shared genetic etiology of psychiatric and gastrointestinal disorders
Source: Commun Med (Lond). 2024 Sep 19;4:180. doi: 10.1038/s43856-024-00607-7 (PMC11413006; doi:10.1038/s43856-024-00607-7)
Supplement: Supplementary file 3 — Description of Additional Supplementary Files [file 43856_2024_607_MOESM3_ESM.pdf]

Description of Additional Supplementary Files

File Name: Supplementary Data 1.
Description: Distributions and associations of parental history of psychiatric disorders and risk of PUD/GERD/IBS/IBD (sample size=4,504,612)

File Name: Supplementary Data 2.
Description: Distributions and associations between same-sex twin's history of psychiatric disorders and risk of PUD/GERD/IBS/IBD (sample size=51,664)

File Name: Supplementary Data 3.
Description: Distributions and associations between full sibling's history of psychiatric disorders and risk of PUD/GERD/IBS/IBD (sample size=3,322,959)

File Name: Supplementary Data 4.
Description: Distributions and associations between full sibling's history of psychiatric disorders (dose-response relationship) and risk of PUD/GERD/IBS/IBD (sample size=3,322,959)

File Name: Supplementary Data 5.
Description: Distributions and associations of parental history of gastrointestinal disorders and risk of SCZ/BPD/MDD/OCD (sample size=4,504,612)

File Name: Supplementary Data 6.
Description: Distributions and associations between same-sex twin's history of gastrointestinal disorders and risk of SCZ/BPD/MDD/OCD (sample size=51,664)

File Name: Supplementary Data 7.
Description: Distributions and associations between full sibling's history of gastrointestinal disorders and risk of SCZ/BPD/MDD/OCD (sample size=3,322,959)

File Name: Supplementary Data 8.
Description: Distributions and associations between full sibling's history of gastrointestinal disorders (dose-response relationship) and risk of SCZ/BPD/MDD/OCD (sample size=3,322,959)

File Name: Supplementary Data 9.
Description: Sex-stratified association of individual history and family history, including parents, same-sex twins, and full siblings, of psychiatric disorders with the risk of PUD/GERD/IBS/IBD

File Name: Supplementary Data 10.
Description: Sex-stratified association of individual history and family history, including parents, same-sex twins, and full siblings, of gastrointestinal disorders with the risk of SCZ/BPD/MDD/OCD

File Name: Supplementary Data 11.
Description: Association of decile of polygenic risk score for psychiatric disorders with

gastrointestinal disorders.

File Name: Supplementary Data 12.
Description: Association of decile of polygenic risk score for gastrointestinal disorders with psychiatric disorders.

File Name: Supplementary Data 13.
Description: The bidirectional causality estimates between psychiatric and gastrointestinal disorders
