# Supplementary material for: A population-based study of familial coaggregation and shared genetic etiology of psychiatric and gastrointestinal disorders
Source: Commun Med (Lond). 2024 Sep 19;4:180. doi: 10.1038/s43856-024-00607-7 (PMC11413006; doi:10.1038/s43856-024-00607-7)
Supplement: Supplementary file 5 — Reporting Summary [file 43856_2024_607_MOESM5_ESM.pdf]

nature portfolio

Corresponding author(s): Shi-Heng Wang

Last updated by author(s): Jun 18, 2024

Reporting Summary

Nature Portfolio wishes to improve the reproducibility of the work that we publish. This form provides structure for consistency and transparency in reporting. For further information on Nature Portfolio policies, see our Editorial Policies and the Editorial Policy Checklist.

Statistics

For all statistical analyses, confirm that the following items are present in the figure legend, table legend, main text, or Methods section.

n/a	Confirmed	
☐	☒	The exact sample size (*n*) for each experimental group/condition, given as a discrete number and unit of measurement
☐	☒	A statement on whether measurements were taken from distinct samples or whether the same sample was measured repeatedly
☐	☒	The statistical test(s) used AND whether they are one- or two-sided<br>*Only common tests should be described solely by name; describe more complex techniques in the Methods section.*
☐	☒	A description of all covariates tested
☐	☒	A description of any assumptions or corrections, such as tests of normality and adjustment for multiple comparisons
☐	☒	A full description of the statistical parameters including central tendency (e.g. means) or other basic estimates (e.g. regression coefficient) AND variation (e.g. standard deviation) or associated estimates of uncertainty (e.g. confidence intervals)
☐	☒	For null hypothesis testing, the test statistic (e.g. *F*, *t*, *r*) with confidence intervals, effect sizes, degrees of freedom and *P* value noted<br>*Give P values as exact values whenever suitable.*
☒	☐	For Bayesian analysis, information on the choice of priors and Markov chain Monte Carlo settings
☒	☐	For hierarchical and complex designs, identification of the appropriate level for tests and full reporting of outcomes
☒	☐	Estimates of effect sizes (e.g. Cohen's *d*, Pearson's *r*), indicating how they were calculated

Our web collection on statistics for biologists contains articles on many of the points above.

Software and code

Policy information about availability of computer code

Data collection	No software was used.
Data analysis	Statistical analyses were performed using IMPUTE2, PLINK 1.90, PRS-CS, PRS-CSx, LD score regression, TwoSampleMR R package, and SAS 9.4.

For manuscripts utilizing custom algorithms or software that are central to the research but not yet described in published literature, software must be made available to editors and reviewers. We strongly encourage code deposition in a community repository (e.g. GitHub). See the Nature Portfolio guidelines for submitting code & software for further information.

Data

Policy information about availability of data

All manuscripts must include a data availability statement. This statement should provide the following information, where applicable:

- Accession codes, unique identifiers, or web links for publicly available datasets
- A description of any restrictions on data availability
- For clinical datasets or third party data, please ensure that the statement adheres to our policy

Publicly available data are available from the following sites: GWAS summary results for psychiatric diseases were downloaded from https://www.med.unc.edu/pgc/; GWAS summary results for PUD were downloaded from https://cnsgenomics.com/content/data; GWAS summary results for GERD were downloaded from http://ftp.ebi.ac.uk/pub/databases/gwas/summary_statistics/GCST90000001-GCST90001000/GCST90000514/; GWAS summary results for IBS were downloaded from http://ftp.ebi.ac.uk/pub/databases/gwas/summary_statistics/GCST90016001-GCST90017000/GCST90016564/; GWAS summary results for IBD were

downloaded from http://ftp.ebi.ac.uk/pub/databases/gwas/summary_statistics/GCST004001-GCST005000/GCST004131/.
The NHIRD used in this study is held by the Taiwan Ministry of Health and Welfare and under controlled access. Researchers interested in accessing the data set can submit an application form to the Ministry of Health and Welfare requesting access. Taiwan Biobank data used in this study is under controlled access. Application to access can be made to the Taiwan Biobank.

Research involving human participants, their data, or biological material

Policy information about studies with human participants or human data. See also policy information about sex, gender (identity/presentation), and sexual orientation and race, ethnicity and racism.

Reporting on sex and gender	The biological sex information was obtained from registry in the NHIRD and self-report in the Taiwan Biobank.
Reporting on race, ethnicity, or other socially relevant groupings	The study samples from the NHIRD and the Taiwan Biobank are Taiwanese of East Asian populations. In the genetic association analyses, principal components analysis was performed for adjusting population stratification.
Population characteristics	Altogether 4,504,612 individuals born between January 1, 1970, and December 31, 1999 were included from the NHIRD. 131,048 individuals with genome-wide genotyping were recruited from the Taiwan Biobank.
Recruitment	The cohort study samples are from the claims database in Taiwan's single-payer compulsory insurance program with extensive coverage rate (99%), thus there is no ascertainment bias. The biobank study samples from the Taiwan Biobank are not representative of the sampling population, and a healthy volunteer selection bias is possible.
Ethics oversight	This study was approved by the Central Regional Research Ethics Committee of the China Medical University, Taichung, Taiwan.

Note that full information on the approval of the study protocol must also be provided in the manuscript.

Field-specific reporting

Please select the one below that is the best fit for your research. If you are not sure, read the appropriate sections before making your selection.

☒ Life sciences ☐ Behavioural & social sciences ☐ Ecological, evolutionary & environmental sciences

For a reference copy of the document with all sections, see nature.com/documents/nr-reporting-summary-flat.pdf

Life sciences study design

All studies must disclose on these points even when the disclosure is negative.

Sample size	Altogether 4,504,612 individuals were included in the nationwide cohort study; 131,048 samples with genome-wide genotyping were included in the biobank study. There was no sample size and power calculation.
Data exclusions	In the quality control process of the biobank study, duplicated samples, non-EAS samples, samples with a missing rate of more than 2%, heterozygosity outliers (exceeding 5 standard deviation) were excluded. To remove cryptic relatedness, one of the study participants was excluded if pair-wise participants with PI-HAT > 0.1875. Finally, 106,796 unrelated participants were included in the analyses.
Replication	The current findings were not replicated in an independent sample.
Randomization	This is an observational study, and randomization is not applicable.
Blinding	This is an observational study, and blinding is not applicable.

Reporting for specific materials, systems and methods

We require information from authors about some types of materials, experimental systems and methods used in many studies. Here, indicate whether each material, system or method listed is relevant to your study. If you are not sure if a list item applies to your research, read the appropriate section before selecting a response.

Materials & experimental systems

n/a	Involved in the study
☒	☐ Antibodies
☒	☐ Eukaryotic cell lines
☒	☐ Palaeontology and archaeology
☒	☐ Animals and other organisms
☒	☐ Clinical data
☒	☐ Dual use research of concern
☒	☐ Plants

Methods

n/a	Involved in the study
☒	☐ ChIP-seq
☒	☐ Flow cytometry
☒	☐ MRI-based neuroimaging
